# Prevalence and risk of complications in untreated patients with adult growth hormone deficiency

**DOI:** 10.1007/s11102-025-01500-9

**Published:** 2025-02-18

**Authors:** Hidenori Fukuoka, Takaaki Endo, Satoshi Tsuboi, Shingo Fujio

**Affiliations:** 1 Division of Diabetes and Endocrinology, Department of Internal Medicine, Kobe University Hospital, Hyogo, Japan; 2 Novo Nordisk Pharma Ltd, Meiji Yasuda Seimei Building, 2-1-1, Marunouchi, Chiyoda-ku, Tokyo, 100-0005 Japan; 3 Department of Neurosurgery, Graduate School of Medical and Dental Sciences, Kagoshima University, Kagoshima, Japan

**Keywords:** Growth hormones, Adult growth hormone deficiency, Prevalence, Complications, Real-world data, Database study

## Abstract

**Purpose:**

Adult growth hormone deficiency (AGHD) increases the prevalence of complications, including metabolic disorder, leading to increased cardiovascular mortality from cardiovascular diseases. However, no large database studies have evaluated AGHD patients without GH replacement therapy (GHRT). We investigated the prevalence of AGHD-related complications in patients without GHRT.

**Methods:**

Patients with AGHD and associated complications were identified from the Medical Data Vision claims database using Japanese local disease codes mapped to ICD-10 codes. The prevalence of AGHD-related complications in 2020 was estimated to compare with the prevalence in the Japanese general population in the latest available year 2020. Risk factors for complications were evaluated by Kaplan-Meier curves and a Cox proportional hazard model.

**Results:**

We identified 8,809 untreated patients with AGHD from April 2008 to September 2022, including 3,430 in 2020. In 2020, the prevalence of complications was higher in the AGHD population adjusted for sex and age than in the Japanese general population, e.g., diabetes mellitus, 9.3% vs. 3.6%; osteoporosis, 4.8% vs. 1.3%; and dyslipidemia, 22.0% vs. 3.9%. Age was a significant risk factor for most complications, and female sex for osteoporosis. Diabetes mellitus was a significant risk factor for dyslipidemia, ischemic heart disease, cerebrovascular disease, and all-cause death.

**Conclusion:**

Untreated patients with AGHD have a higher prevalence of metabolic complications than the general population despite no difference in their related risk factors. Given the low use of GHRT in this study, comprehensive treatment approaches that include GHRT need to be considered to alleviate the risk of complications.

**Supplementary Information:**

The online version contains supplementary material available at 10.1007/s11102-025-01500-9.

## Introduction

Growth hormone (GH), which is mainly secreted from the pituitary somatotroph, promotes bone and muscle development in childhood, as well as plays a crucial role in regulating metabolism in adulthood [1]. Adult GH deficiency (AGHD) can be caused by an acquired impairment in the secretion of this peptide as a result of a sellar mass disorder, perisellar surgery, or radiation, as well as by traumatic brain injury and subarachnoid hemorrhage [1–4]. Recently, autoimmune cytotoxicity specific to pituitary-specific transcription factor 1 (PIT-1) lineage cells, including somatotrophic cells, was also identified as a cause of acquired GH deficiency (GHD) as a paraneoplastic syndrome [5].

Patients with AGHD frequently have reduced quality of life (QoL) because of subjective symptoms such as fatigue and impaired concentration [6, 7], but AGHD is also known to be associated with various metabolic disorders, including increased prevalence of dyslipidemia, diabetes mellitus (DM), hypertension, osteoporosis, and non-alcoholic steatohepatitis (NASH) [8]. Metabolic abnormalities are associated with increased mortality, mainly due to cardiovascular events, which can lead to a shortened life expectancy [9–11].

In patients diagnosed with AGHD, GH replacement therapy (GHRT) is expected to alleviate subjective symptoms and improve metabolic and other AGHD-related complications [8]. However, there could be a substantial number of patients with AGHD who do not receive GHRT [12]. These untreated patients have a higher risk of AGHD-related complications, so it is important to evaluate complications in this population.

Although registry studies have been performed previously in patients with AGHD [13], no large database studies have examined patients with AGHD and without GHRT (GHRT-naïve). Therefore, we performed a retrospective database study in GHRT-naïve Japanese patients with AGHD (diagnosed as per the MDV claims database), with a primary objective of investigating the prevalence of AGHD-related complications such as diabetes mellitus, osteoporosis, and dyslipidemia in these patients. Other objectives included identifying the significant risk factors associated with these complications using real-world data and comparing the prevalence of these complications in patients with AGHD to that in the general population, in order to assess the health risks associated with untreated AGHD.

## Materials and methods

### Study design

This was a real-world, retrospective database study that used the MDV claims database (Medical Data Vision Co., Ltd., Tokyo, Japan) and evaluated data from April 2008 to September 2022. MDV is a hospital-based database and, as one of the largest claims databases in Japan, covers 26% of acute care hospitals. All data in the database are anonymized. In this study, we investigated the prevalence of and risk factors for AGHD-related complications by using Kaplan-Meier curves and Cox proportional hazards models. The study was approved by the MINS Research Ethics Committee (MINS-REC-230201; approved on January 19, 2023).

### Patient identification

To identify the database-defined patients with AGHD from the claims database, we used disease codes expected to include AGHD (Online Resource, Table S1), such as hypopituitarism and pituitary hormone deficiency, in addition to codes for AGHD itself. We took this approach because patients with AGHD are not necessarily recorded with a disease code for AGHD, but rather with a disease code for other conditions. The index date was defined as the date of first diagnosis of the disease, and end of the follow-up period was the date of the patients’ last record in the database. Patients aged 18 or more at the index date were included. To avoid misclassification, patients with documented anterior pituitary malfunction were selected by excluding those not receiving pituitary hormone replacement therapy other than desmopressin. Exclusion criteria were as follows: Patients with incomplete or missing data, patients who had received GHRT, and patients diagnosed with isolated adrenocorticotropic hormone, thyroid stimulating hormone, or gonadotropin deficiency were excluded because they may not have had GHD. However, patients diagnosed with isolated or severe GHD were included.

**Fig. 1:**
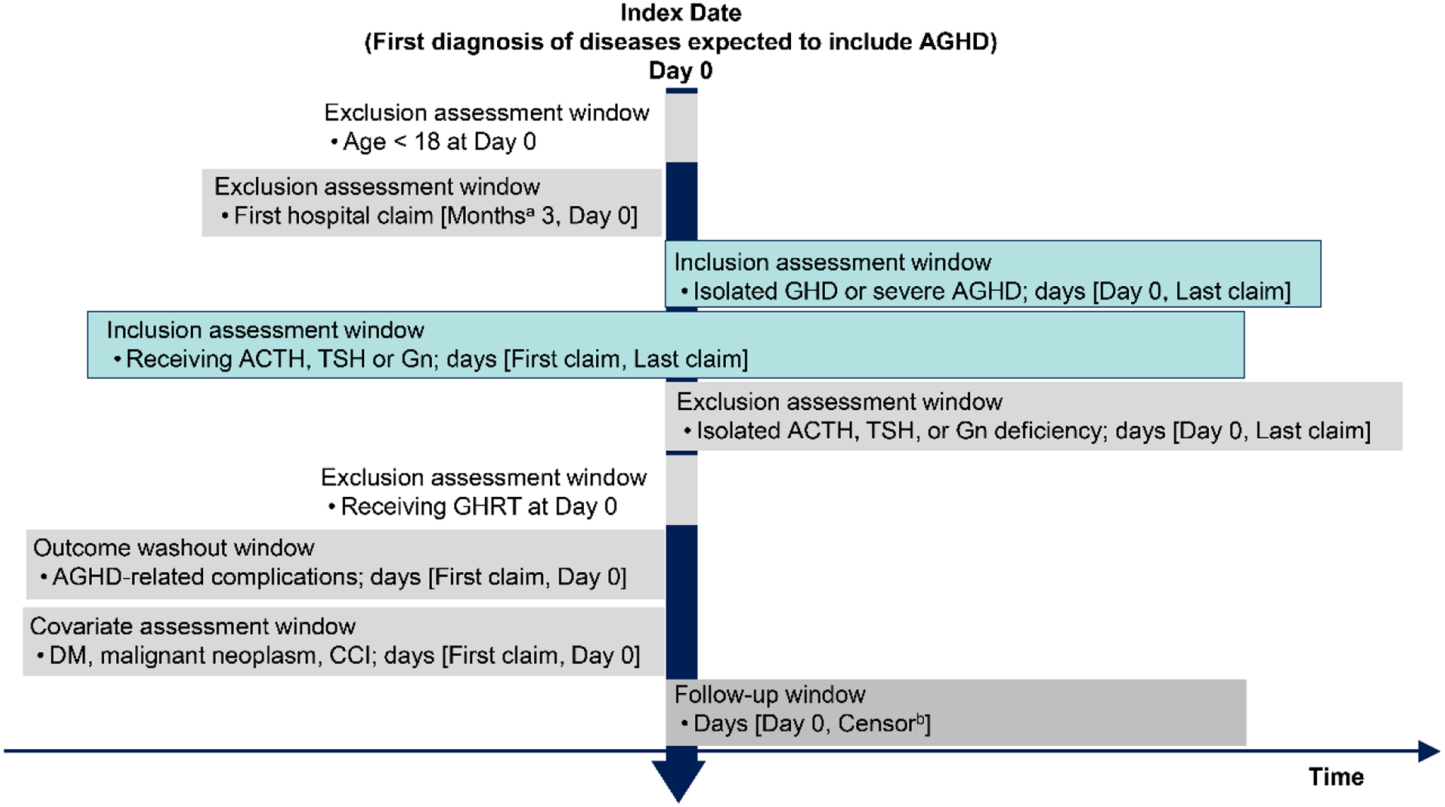
Study design ^a^Claims are collected from hospitals on a monthly basis. ^b^Earliest onset of AGHD-related complications or the last claim of the patient. ACTH, adrenocorticotropic hormone; AGHD, adult growth hormone deficiency; CCI, Charlson comorbidity index; DM, diabetes mellitus; GHD, growth hormone deficiency; Gn, gonadotropin; GHRT, growth hormone replacement therapy; TSH, thyroid stimulating hormone

Although most patients would be expected to be diagnosed with AGHD during sellar-related events, some may be diagnosed only after being referred to a specialist for treatment of complications because AGHD presents with non-specific symptoms. As accurate diagnosis of AGHD using the International Classification of Diseases, Tenth Revision (ICD-10) codes without GH testing or verification was challenging, we refined our identification process to include the most likely cases of AGHD, focusing on the most specific disease codes, with help from experts in the AGHD field. Because patients could be diagnosed with AGHD at their first visit after being transferred from other hospitals, we did not set a certain duration of the observable period before the index date as an inclusion criterion. However, to ensure that patients were new to the hospital and had no history of visits, their index date had to be more than 3 months after the month in which the MDV database started to collect data from the hospital (Fig. 1). For comparison with general population, data were obtained from national surveys for Japanese population aged 18 years or more.

### Outcomes

The prevalence of complications in patients with database-defined AGHD without GHRT was estimated by dividing the number of patients who developed complications by the total number of patients. Data from the 2020 national census were used as a reference to compare the real-world prevalence in the MDV database with that in the Japanese general population. We estimated the prevalence in 2020 and adjusted it for age and sex because the latest national statistics [14, 15] on the number of patients, including those with AGHD-related complications such as DM, were for 2020. In the analysis of prevalence, an adult was defined as someone aged 20 years or over because the national statistics are reported in 5-year age increments.

AGHD-related complications were defined according to ICD-10 and local disease codes, as well as by World Health Organization Anatomical Therapeutic Chemical codes of prescriptions for those diseases where appropriate (Online Resource, Table S2).

### Statistical analysis

A formal sample size calculation was not conducted for this study, as data for all eligible patients available in this large database were collected. The background characteristics of patients were summarized. Continuous variables were reported as mean, standard deviation (SD), median, interquartile ranges, minimum, and maximum. Categorical variables were summarized as frequencies and percentages of the study population. No imputation for missing values was performed, since if the data for any variables were unavailable, the patients were excluded from analyses.

Kaplan-Meier curves of AGHD-related complications were created to visually assess the time and frequency of the onset of AGHD-related complications in patients with AGHD. The cohort of untreated patients with AGHD may have included those who could not receive GHRT because of complications such as DM and malignant neoplasm that contraindicated its use, so Kaplan-Meier curves were drawn for patients with and without those complications. A Cox proportional hazard model was also used to evaluate the risk factors for the onset of AGHD-related complications. The multivariate Cox regression analyses were performed adjusting for confounding variables such as age, sex, and comorbidities to evaluate the independent association of purported risk factors with the complications. Covariates of Cox regression were (1) age at index date; (2) sex; (3) history of any malignant neoplasm; (4) history of DM; and (5) Charlson comorbidity index (for all-cause death) [16, 17]. As a sensitivity analysis to test the robustness of our results, we analyzed cases where an AGHD-related complication did not require use of any concomitant medication and performed a subgroup analysis of cases of severe AGHD. For patients with a recorded body mass index (BMI), we also performed an analysis with BMI as a covariate.

All statistical analyses were performed with R (version 4.2.1, R Foundation, Vienna, Austria). Statistical significance was considered at a two-sided *P* value of less than 0.05.

## Results

### Background of patients with AGHD

We identified 8,809 eligible patients with AGHD, including those who received GHRT, as shown in Online Resource, Fig. S1. Patient characteristics are summarized in Table 1. Only 8.1% (717) had received GHRT, 56.5% (4,977) were women, and the mean ± SD age at initial diagnosis was 55.7 (18.4) years.

**Table 1:** Characteristics of patients with AGHD (*N* = 8,809) included from April 2008 to September 2022 in the MDV claims database

Category		Value	Mean ± SD
Age, y			55.7 ± 18.4
	Minimum	18	
	Median	57	
	Maximum	100	
**Category**		**n**	**%**
GHRT	Yes	717	8.1%
	No	8,092	91.9%
Sex	Male	3,832	43.5%
	Female	4,977	56.5%
Type of hospital	Small (< 200 beds)	301	3.4%
	Medium (≥200 to < 500 beds)	3,294	37.4%
	Large (≥500 beds)	5,214	59.2%
Pituitary neoplasm	Yes	3,066	34.8%
Type of neoplasm^*a*^	Malignant craniopharyngioma	4	0.1%
	Malignant pituitary tumor	12	0.4%
	Pituitary adenoma	1,630	53.2%
	Benign craniopharyngioma	66	2.2%
	Pituitary tumor	1,830	59.7%
	Craniopharyngioma	391	12.8%
Number of pre-operational GH stimulation tests	None	7,162	81.3%
	1	1,216	13.8%
	2	348	4.0%
	3	49	0.6%
	4 or more	34	0.4%
Pituitary surgeries	Yes	642	7.3%
Type of surgery^*b*^	Endoscopic trans-nasal pituitary tumor extirpation	371	57.8%
	Trans-nasal pituitary tumor extirpation	150	23.4%
	Intracranial tumor extirpation (other than pineal tumor)	132	20.6%
Number of post-operational GH stimulation tests	None	369	57.5%
	1	185	28.8%
	2	73	11.4%
	3	10	1.6%
	4 or more	5	0.8%
Radiation therapies	Yes	396	4.5%
Type of radiation therapy^*c*^	External irradiation	323	81.6%
	Radiation therapy management	255	64.4%
	Blood irradiation	81	20.5%
	Whole body irradiation	4	1.0%
	Sealed small source radiation therapy	1	0.3%
	Electromagnetic thermotherapy	0	0.0%

AGHD, Adult growth hormone deficiency; DM, Diabetes mellitus; GH, Growth hormone; GHRT, GH replacement therapy; MDV, Medical Data Vision

^*a*^Patients with each tumor type as a percentage of patients with any pituitary tumor (multiple answers allowed)

^*b*^Patients who underwent each type of surgery as a percentage of patients who underwent pituitary surgery (multiple answers allowed)

^*c*^Patients who received each type of radiation as a percentage of patients who received any radiation therapy (multiple answers allowed)

**Fig. 2:**
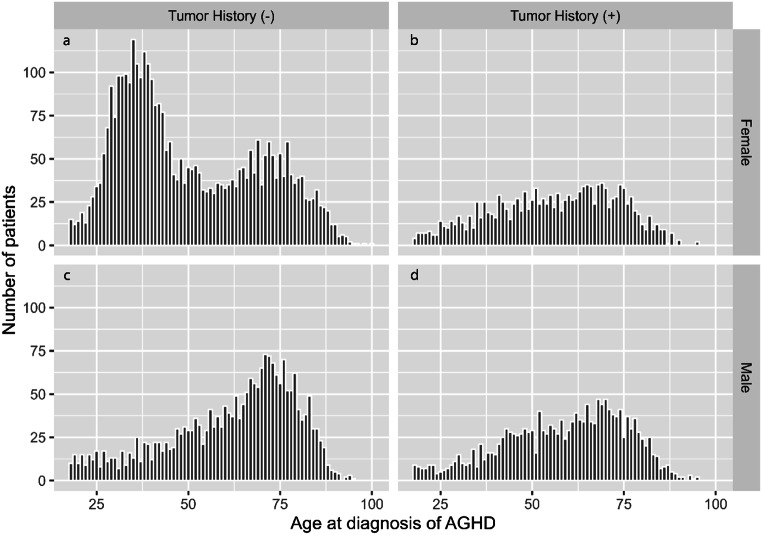
Histogram of age at diagnosis of adult growth hormone deficiency by sex and history of pituitary tumor. (**a**) females without tumor (**b**) females with tumor (**c**) males without tumor (**d**) males with tumor. AGHD, adult growth hormone deficiency

A histogram of age at diagnosis by sex and history of pituitary tumor is shown in Fig. 2. There was a bimodal distribution only in women without pituitary tumor.

### Prevalence of AGHD-related complications in patients without GHRT

To compare the prevalence of AGHD-related complications in the MDV database with that in the Japanese general population, we compared GH-naïve patients with AGHD included in the database in 2020, regardless of complications (3,430 out of 8,809 such patients overall), with the latest published statistics in the Japanese general population for 2020. The estimated prevalences are shown in Table 2. The prevalence of complications in the GH-naïve AGHD population adjusted for sex and age was higher than that of Japanese general population.

**Table 2:** Estimated prevalence of AGHD-related complications in 2020

Complications	Number of patients in MDV with complications	Prevalence (%)	
		MDV database^a^, %	General population^b^, %
All	3,430		
DM	351	9.30	3.60
Glucose intolerance	133	4.12	NR^*c*^
Dyslipidemia	797	22.02	3.90
Fatty liver	24	0.68	NR^*d*^
NASH/NAFLD	4	0.13	NR^*e*^
Liver cirrhosis	15	0.34	0.10
Osteoporosis	174	4.76	1.32
Fracture	135	4.00	0.88
Ischemic heart disease	125	2.98	1.24
Cerebrovascular disease	129	3.36	1.69
Depression	121	3.37	1.66

AGHD, Adult growth hormone deficiency; DM, Diabetes mellitus; MDV, Medical Data Vision; NASH, Nonalcoholic steatohepatitis; NAFLD, Nonalcoholic fatty liver disease; NR, Not reported

^*a*^Adjusted by sex and age to match the Japanese general population as recorded in the national census 2020 [14, 15]

^b^Estimated by dividing the number of patients with each complication estimated in patient survey 2020 [15] by the number of the Japanese population aged 18 or older in the national census 2020 [14]

^*c*^Mean HbA1c value of 5.8% in men and 5.7% in females as reported per 2019 National Health and Nutrition Survey [18]

^*d*^The number of patients with fatty liver is reported to be more than 20 million [19] and using the total population of the national census 2020, the prevalence could be estimated at 15.9%

^*e*^Prevalence of NAFLD is reported to be 24.7–41.0% in men and 9.7–17.7% in women [20, 21]. If 10–20% of NAFLD is NASH [22], the prevalence of NASH is 3.2–8.2% in men and 0.9–3.5% in women

### Risk factors for AGHD-related complications

The number of patients with each AGHD-related complication pre- and post-index date is shown in Online Resource, Table S3. While dyslipidemia was the most frequent complication before the index date (13.9%; 1,126/8,092), 915 (13.1%) out of the 6,966 patients without dyslipidemia at index date newly developed the disease after the index date. On the other hand, few cases of liver disease (i.e., fatty liver, NASH/nonalcoholic fatty liver disease [NAFLD], and liver cirrhosis) were detected.

**Fig. 3:**
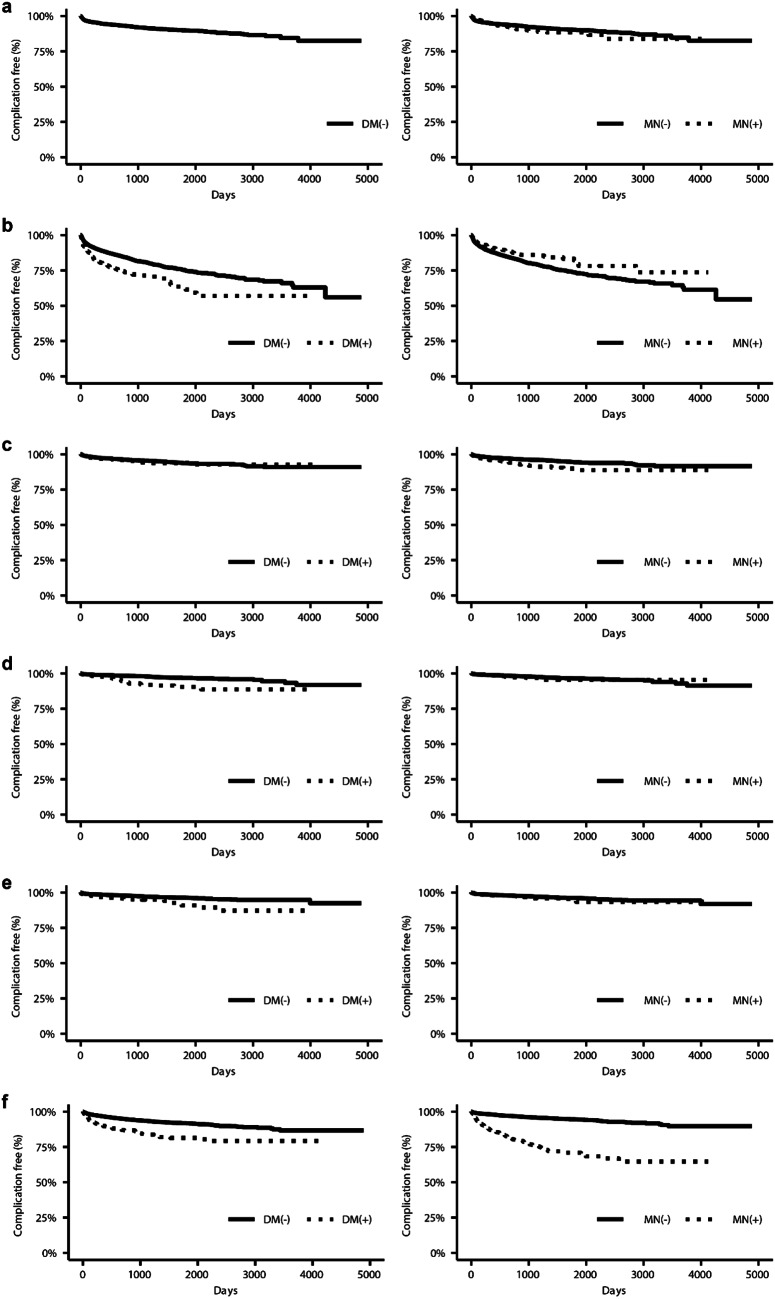
Kaplan-Meier curve for onset of adult growth hormone deficiency-related complications by history of diabetes mellitus (DM; left) and malignant neoplasm (MN; right) (**a**) DM (**b**) dyslipidemia (**c**) osteoporosis (**d**) ischemic heart disease (**e**) cerebrovascular disease (**f**) all-cause death

The Kaplan-Meier plots of AGHD-related complications are shown in Fig. 3. Median survival time was not reached for all complications. Rates of complications were similar in patients with and without malignant neoplasm, but patients with DM had a higher incidence of dyslipidemia, ischemic cardiac disease, and cerebrovascular disease than patients without DM. The result of Cox regression for each complication is summarized in Online Resource, Table S4. The hazard ratio for age was significantly higher than 1 for all complications except glucose intolerance and liver disease, and that for being female was also significantly higher than 1 for osteoporosis and fracture. DM was a significant risk factor for dyslipidemia, ischemic heart disease, cerebrovascular disease, and all-cause death. A sensitivity analysis with an alternative definition of AGHD-related complications defined without the requirement of use of concomitant medication showed similar results as described above (Data not shown). A subgroup analysis of patients with severe AGHD did not identify any significant risk factors other than DM for dyslipidemia, probably because of the small number of cases (Data not shown). When BMI was included as a covariate of Cox regression, it was found to be a significant risk factor for DM and dyslipidemia, fracture, and all-cause death (Data not shown).

## Discussion

To the best of our knowledge, this was the first database study to assess the prevalence of AGHD-related complications in GH-naïve patients with AGHD (diagnosis per claims database).

Women accounted for 56.5% of patients and showed two peaks in the histogram of age at diagnosis of AGHD, at ages 30 to 40 years and 60 to 70 years, whereas men showed only the latter peak. The bimodal distribution in women was found only in those without a history of pituitary tumor, and the women had a diagnosis of AGHD. Hypopituitarism can be easy to recognize in women because of menstrual abnormalities, whereas it can be more difficult in men, which could explain why no peak was seen in men at age 30 to 40 years. Another possible reason is that pregnancy-related hypopituitarism, including autoimmune hypophysitis and Sheehan’s syndrome, may be associated with this first peak.

About 17.4% of patients from the AGHD study cohort had malignant tumors. Although this implied a higher mortality risk in this subgroup compared to the general population, this reflected the actual conditions of AGHD patients from the real-world scenario and was in line with our primary goal.

In this study, we included patients with AGHD who were diagnosed just after being transferred from other hospitals and no data were available on previous pituitary surgery or radiation therapy. Thus, the proportion of patients with pituitary tumor, pituitary surgery, and radiation therapy may have been underestimated. Methodological differences between our and previous studies may explain some of the differences observed in these proportions [23].

The prevalence of AGHD-related metabolic complications in GH-naïve patients with AGHD was higher than that of metabolic complications in the general population. As AGHD causes metabolic disorder and an altered lipid profile [24], it is not surprising that dyslipidemia was the most frequent complication, with the greatest difference in prevalence between patients with AGHD and the general population (22.0% vs. 3.9%).

Estimated prevalences of complications were similar to or lower than those in previous studies. For instance, Mo et al. reported a prevalence of 14.0% for DM in GH-naïve patients with AGHD, which aligned closely with the prevalence in our study (9.3%) [25]. However, they found a higher prevalence of osteoporosis and depression than we did (10.7% vs. 4.8% and 16.1% vs. 3.4%, respectively), which could be due to a difference in study designs or advances in medical care. They also reported that 42.5% of untreated patients used lipid-lowering medication, which is higher than the prevalence of dyslipidemia (22.0%) in our study. The prevalence of these complications could also be underestimated in our study because of the nature of a database study. Incidences of myocardial infarction and angina in Japanese patients with AGHD were 1.2% and 2.8%, respectively, while those of cerebral infarction and cerebral hemorrhage were 3.7% and 0.8%, respectively [26], comparable to our results for ischemic heart disease (2.98%) and cerebrovascular disease (3.36%). Based on the patient demographics and complications observed in this study, we consider our findings to be representative of Japanese patients with AGHD.

Nonetheless, the prevalence of glucose intolerance and liver disease may have been especially underestimated because we could only identify complications recorded in the claims database. In 2020, the estimated prevalence of AGHD-related complications in patients with AGHD was higher than that in the general population from the Japanese national census. The prevalence of DM in our study (9.3%) was higher than that in the general population (4.2%) assessed from hospital-based data [27]. Furthermore, glucose intolerance was less common in patients with AGHD (4.1%) than in the general population with mean HbA1c value of 5.8% in men and 5.7% in females as reported per 2019 National Health and Nutrition Survey [18]. Our study found fewer cases of fatty liver (0.7%) and NASH/NAFLD (0.1%) than a previous study in the general population (27.7% for fatty liver, 23.7% for NAFLD) [28]. Liver disease is particularly prone to underreporting because the diagnostic procedures often require abdominal ultrasonography, computed tomography, magnetic resonance imaging, and liver biopsies, which are not always conducted in real-world clinical settings. Furthermore, because no established treatments are available for liver disease, medical costs may not be incurred by hospitals; hence, liver disease may not be recorded in the claims database. Terai et al. analyzed the MDV database and reported a prevalence of 1.93% for NASH/NAFLD in Japanese adults on a diagnosis basis [29]. On the other hand, Yoneda et al. estimated a much higher prevalence of 9.2% for NAFLD with the Fatty Liver Index prediction model by using medical check-up records in the JMDC database [30]. Therefore, the prevalence of liver disease may have been lower in this study because we could only identify complications recorded in the claims database, which may not have captured all cases.

We visualized AGHD-related complications with Kaplan-Meier curves. Although some previous studies focused on specific complications, to the best of our knowledge, ours was the first study to address a broad range of complications in patients with untreated AGHD. Unfortunately, in Japan, GHRT was contraindicated in patients with AGHD, not only those with malignant neoplasm but also in those with DM, up to 2022 including our study period. We prepared a Kaplan-Meier curve of AGHD both with and without these complications and found that the incidence of most complications was higher in patients with AGHD and DM or malignant neoplasm. However, the incidence of dyslipidemia was lower in patients with AGHD and malignant neoplasm than in those without. As dyslipidemia is one of the risk factors for malignancy, patients with malignant neoplasm could have a higher prevalence of dyslipidemia at index date than those without, which means fewer patients with malignancy would be newly diagnosed as dyslipidemia.

Although the effect of confounding variables cannot be completely ruled out, we explored the risk factors for complications using a multivariate Cox proportional hazard model adjusting for probable confounders. Although the prevalence of complications was higher in patients with AGHD than in the general population, the risk factors were similar in both groups. Age was a significant risk factor for most complications; being female, for osteoporosis and fracture; and DM, for dyslipidemia, ischemic heart disease, cerebrovascular disease, and all-cause death. The results of Cox regression captured the characteristics of each disease well, indicating that we used relevant definitions to identify each disease from the database.

Although it is crucial to acknowledge the complications associated with AGHD, it is also important to recognize the promising therapies available to mitigate these risks. GHRT is reported to ameliorate the risk of complications in patients with AGHD, e.g., studies reported reduced cardiovascular risk [31] and standardized mortality ratio [32] in patients with AGHD receiving GHRT. Improvement of body composition, lipid profile [33], and bone mineral density [34] were also described, and GHRT was found to prevent fractures [25]. Dyslipidemia, understandably the most frequent complication in our study, is not only one of the metabolic complications, but is also considered a significant contributor of cardiovascular risk among untreated GH-deficient adults today. Appropriate GHRT can positively impact the lipid profile [35, 36]. Furthermore, as assessed by the Adult Hypopituitarism Questionnaire, improved QoL was also reported in patients with AGHD [37].

In this study, we showed the real-world prevalence of AGHD-related complications in patients with untreated AGHD. It is noteworthy that only 8.1% of patients with AGHD received GHRT, a remarkably low percentage given the established benefit of GHRT. The low intervention rate suggests an unmet need for comprehensive treatment approaches in patients with AGHD; efforts are also needed to reduce the risk factors for related complications, to improve patient outcomes and quality of life. Taken together, the results of this database study and previous reports on each complication help us to provide more evidence-based guidance for the management of patients with AGHD. Avenues for further research include improved diagnostic criteria and patient management strategies for ensuring long-term benefits of GHRT.

This study has several limitations generally inherent to claims database research and specifically to the MDV database used to identify AGHD patients. Patients with AGHD and its associated complications were identified using ICD-10 codes, laboratory tests, and available medical records from the database. However, specific details essential for a definitive diagnosis of AGHD, such as GH secretion stimulation test data, IGF-I, medication details are often not captured in MDV, leading to potential misidentification of diseases. Indeed, this is a common limitation in database research [38], especially in therapeutic areas of rare diseases. MDV being a hospital-based claims database, patients cannot be traced if they change hospital because of relocation, and data on treatments not covered by insurance are not included. Further, some differences in the prevalence of certain complications with higher prevalences among the AGHD patients vs. those in the general population may be attributable to the way in which the data were collected: while the data on the AGHD patients were captured from the MDV database, the general population data were captured using the national census. Thus, the possibility of specialized follow-up and disease-specific testing conducted by endocrinologists managing AGHD leading to a higher detection of complications in the AGHD cohort cannot be denied. Nevertheless, we believe that our results provide the overall prevalence and impact of complications in a broader healthcare context by contrasting the data of an AGHD cohort vs. the general population.

Additionally, it should be noted that unlike rigorous comparative data from clinical trials, these real-world data are supportive in nature and may have limited generalizability. Despite these limitations, the study provides valuable insights into the prevalence and impact of complications. The precise identification of diseases and the establishment of comparison cohorts are critical considerations for future database research. Further discussions on the quality of databases and the refinement of analytical methodologies in database research will enhance its reliability.

## Conclusions

This study with real-world data from large number of patients as well as general population provides valuable insights into the health risks of untreated AGHD. The prevalence of AGHD-related metabolic complications is higher in GH-naïve patients with AGHD than the prevalence of metabolic complications in the general population, but the risk factors for those complications are similar to those in the general population. These findings and the low use of GHRT in patients with AGHD emphasize the importance of early diagnosis and indicate that a comprehensive treatment approach that includes GHRT is essential to alleviate the risk of complications associated with AGHD.

## Electronic supplementary material

Below is the link to the electronic supplementary material.

Supplementary Material 1

## Data Availability

Restrictions apply to the availability of some or all data generated or analyzed during this study to preserve patient confidentiality or because they were used under license. The corresponding author will on request detail the restrictions and any conditions under which access to some data may be provided.
