# Supplementary material for: Prevalence and risk of complications in untreated patients with adult growth hormone deficiency
Source: Pituitary. 2025 Feb 18;28(2):32. doi: 10.1007/s11102-025-01500-9 (PMC11836217; doi:10.1007/s11102-025-01500-9)

**SUPPLEMENTARY SECTION**

**Title:** Prevalence and risk of complications in untreated patients with adult growth hormone deficiency

**Journal:** Pituitary

**Authors:** Hidenori Fukuoka^1^, Takaaki Endo^2^, Satoshi Tsuboi^2^, and Shingo Fujio^3^

^1^Division of Diabetes and Endocrinology, Department of Internal Medicine, Kobe University Hospital, Hyogo, Japan

^2^Novo Nordisk Pharma Ltd., Tokyo, Japan

^3^Department of Neurosurgery, Graduate School of Medical and Dental Sciences, Kagoshima University, Kagoshima, Japan

**Corresponding author:**

Takaaki Endo,

Novo Nordisk Pharma Ltd., Meiji Yasuda Seimei Building, 2-1-1, Marunouchi, Chiyoda-ku, Tokyo 100-0005, Japan

Email: [tend@novonordisk.com](mailto:tend@novonordisk.com)

Tel: +81-80-5479-3287

**Table S1 Definition of possible diseases causing adult growth hormone deficiency**

| **ICD-10 codes** | **Disease code*^a^*** | **Disease name** |
| --- | --- | --- |
| E230 | 2532015 | Panhypopituitarism |
| E230 | 8846213 | Multiple pituitary hormone deficiency |
| E230 | 8831247 | Hypopituitarism |
| E230 | 8835937 | Isolated growth hormone deficiency |
| E230 | 8836822 | Secondary hypopituitarism |
| E230 | 8842944 | Growth hormone deficiency dwarfism |
| E230 | 8844069 | Growth hormone hyposecretion |
| E230 | 8846161 | Severe adult growth hormone deficiency |
| E230 | 8846171 | Adult growth hormone deficiency |
| E230 | 8846213 | Compound pituitary hormone deficiency |
| E230 | 8849746 | Acquired hypopituitarism |
| E893 | 8835323 | Postprocedural hypopituitarism |
| E893 | 8834748 | Post-irradiation hypopituitarism |

ICD-10, International Classification of Diseases- Tenth Revision.

*^a^*Local disease code used in Japanese claims database.

**Table S2 Definition of adult growth hormone deficiency-related complications**

| **Category** | **ICD-10 code (disease code***^a^***)** | **Prescriptions (WHO-ATC)** |
| --- | --- | --- |
| Cancers/Malignant neoplasms | C00-C97  D00-D09 | - |
| DM | E11 | Antidiabetic agent (A10) |
| Glucose intolerance | R73 | - |
| Dyslipidemia | E78 | Antilipidemic agent (C10) |
| Fatty liver | K701 | Bile acids (A05AA02) |
| NASH/NAFLD | K758 (8843497)  K760 (8850319) |  |
| Liver cirrhosis | K703, K74 |  |
| Osteoporosis | M80-M82 | Osteoporosis drugs (M05B) |
| Fracture | SX2, T02, T08, T10, T12 | - |
| Ischemic heart disease | I20-I25 | Antiplatelet agent (B01AC) |
| Cerebrovascular disease | I60-I69 |  |
| Depression | F32, F33 | Psychotropic medications including antidepressant  (N06A, N05B, N05C) |

DM, diabetes mellitus; ICD-10, International Classification of Diseases-Tenth Revision; NASH, nonalcoholic steatohepatitis; NAFLD, nonalcoholic fatty liver disease; WHO-ATC, World Health Organization Anatomical Therapeutic Chemical code.

*^a^*Local disease code used in Japanese claims database.

**Table S3 Number of patients with AGHD with and without related complications**

| **Complication** | **Without complication*^a^*, n (%)** | **With complication*^b^*, n (%)** | | **Eligible for Kaplan-Meier analysis*^c^*, n (%)** |
| --- | --- | --- | --- | --- |
|  |  | **Pre index** | **Post index** |  |
| Dyslipidemia | 6,051 (74.8) | 1126 (13.9) | 915 (11.3) | 6,966 (86.1) |
| Glucose intolerance | 7,028 (87.9) | 897 (11.1) | 167 (2.1) | 7,195 (88.9) |
| DM | 6,884 (85.1) | 739 (9.1) | 469 (5.8) | 7,353 (90.9) |
| Fracture | 7,502 (92.7) | 320 (4.0) | 270 (3.3) | 7,772 (96.0) |
| Osteoporosis | 7,528 (93.0) | 299 (3.7) | 265 (3.3) | 7,793 (96.3) |
| Cerebrovascular disease | 7,603 (94.0) | 299 (3.7) | 190 (2.3) | 7,793 (96.3) |
| Ischemic heart disease | 7,640 (94.4) | 296 (3.7) | 156 (1.9) | 7,796 (96.3) |
| Depression | 7,603 (94.0) | 282 (3.5) | 204 (2.5) | 7,810 (96.5) |
| Liver cirrhosis | 8,017 (99.1) | 43 (0.5) | 32 (0.4) | 8,049 (99.5) |
| Fatty liver | 8,032 (99.3) | 29 (0.4) | 31 (0.4) | 8,063 (99.6) |
| NASH/NAFLD | 8,079 (99.1) | 5 (0.1) | 8 (0.1) | 8,087 (99.9) |

AGHD, adult growth hormone deficiency; DM, diabetes mellitus; NASH, nonalcoholic steatohepatitis; NAFLD, nonalcoholic fatty liver disease.

^a^Without complication means the number of patients who did not have AGHD-related complications during the observational period.

^b^With complication means the number of patients who had AGHD-related complications during the observational period.

^c^Eligible to Kaplan-Meier indicates the number of patients targeted in the Kaplan-Meier curve (without complication + with complication post index).

**Table S4 Hazard ratio for each adult growth hormone deficiency-related complication**

|  | **Hazard ratio [95% CI]** | | | | |
| --- | --- | --- | --- | --- | --- |
|  | **Sex*^a^*** | **Age** | **DM** | **Malignant neoplasm** | **CCI*^b^*** |
| DM | 0.88 [0.73–1.07] | 1.02 [1.01–1.02]* | - | 1.00 [0.79–1.28] | - |
| Glucose intolerance | 0.70 [0.51–0.96]* | 1.00 [0.99–1.01] | - | 0.98 [0.63–1.51] | - |
| Dyslipidemia | 0.78 [0.68–0.89]* | 1.03 [1.02–1.03]* | 1.55 [1.25–1.93]* | 0.53 [0.43–0.65]* | - |
| Fatty liver | 0.87 [0.42–1.81] | 1.00 [0.98–1.03] | 1.81 [0.68–4.81] | 1.80 [0.78–4.18] | - |
| NASH/NAFLD | 6.42 [0.77–53.40] | 1.03 [0.99–1.08] | 1.48 [0.18–12.29] | 0.73 [0.09–6.08] | - |
| Liver cirrhosis | 0.53 [0.26–1.11] | 1.00 [0.98–1.02] | 0.90 [0.27–2.99] | 1.97 [0.88–4.40] | - |
| Osteoporosis | 1.31 [1.02–1.69]* | 1.04 [1.03–1.05]* | 0.89 [0.59–1.34] | 1.59 [1.20–2.10]* | - |
| Fracture | 1.50 [1.18–1.93]* | 1.10 [1.05–1.07]* | 1.40 [0.98–1.94] | 1.20 [0.92–1.66] | - |
| Ischemic heart disease | 0.74 [0.54–1.03] | 1.04 [1.03–1.05]* | 2.39 [1.61–3.54]* | 0.86 [0.56–1.30] | - |
| Cerebrovascular disease | 1.06 [0.79–1.41] | 1.06 [1.04–1.07]* | 1.63 [1.11–2.41]* | 0.79 [0.54–1.16] | - |
| Depression | 1.19 [0.89–1.59] | 1.01 [1.00–1.02]* | 1.01 [0.63–1.61] | 1.76 [1.27–2.43]* | - |
| All-cause death | 0.99 [0.83–1.19] | 1.04 [1.04–1.05]* | 1.40 [1.11–1.76]* | 2.16 [1.65–2.82]* | 1.13 [1.09–1.17]* |

*Significant at 5% level of significance.
CI, confidence interval; CCI, Charlson comorbidity index; DM, diabetes mellitus; NASH, nonalcoholic steatohepatitis; NAFLD, nonalcoholic fatty liver disease.

Rows are independent variables, and columns are covariates of Cox regression.

*^a^*Hazard ratio in female patients relative to male patients.

*^b^*CCI was included as a covariate only for all-cause death.

**Fig. S1** Patient flow

^a^Pituitary hormones other than vasopressin includes ACTH, TSH, Gn, and GH.

ACTH, adrenocorticotropic hormone; AGHD, adult growth hormone deficiency; Gn, gonadotropin; GH, growth hormone; GHD, growth hormone deficiency; GHRT, growth hormone replacement therapy; MDV, Medical Data Vision; TSH, thyroid stimulating hormone.


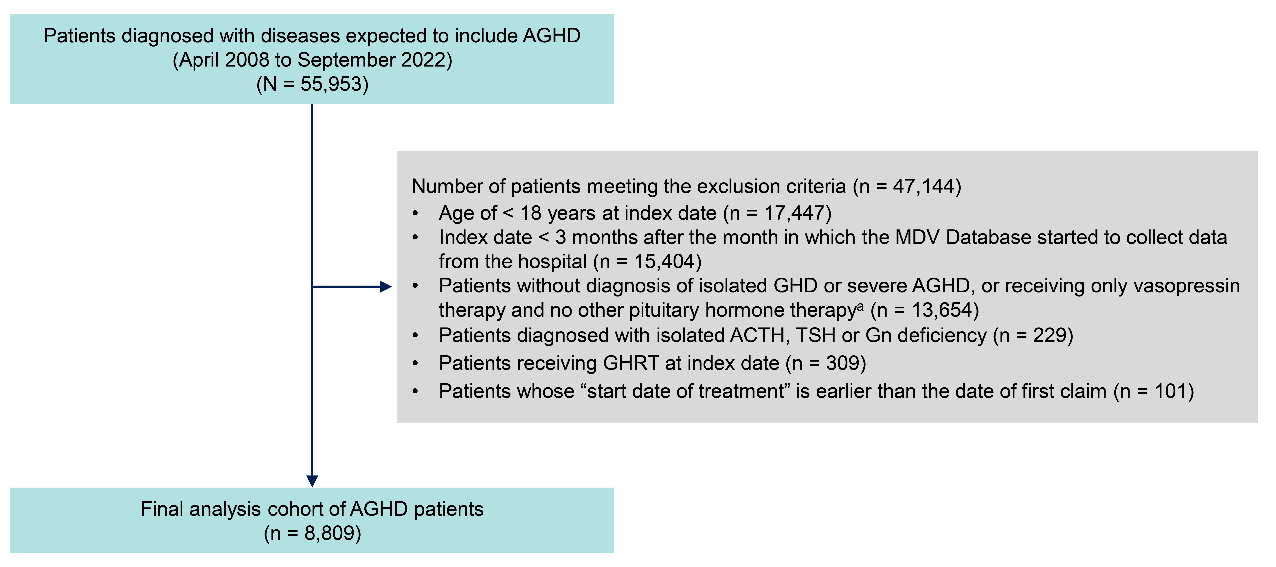

Supplement: Supplementary file 1 — Supplementary Material 1 [file 11102_2025_1500_MOESM1_ESM.docx]
